# Detecting Genetic Interactions for Quantitative Traits Using *m*-Spacing Entropy Measure

**DOI:** 10.1155/2015/523641

**Published:** 2015-08-03

**Authors:** Jaeyong Yee, Min-Seok Kwon, Seohoon Jin, Taesung Park, Mira Park

**Affiliations:** ^1^Department of Physiology and Biophysics, Eulji University, Daejeon, Republic of Korea; ^2^Department of Bioinformatics, Seoul National University, Seoul, Republic of Korea; ^3^Department of Informational Statistics, Korea University, Jochiwon, Republic of Korea; ^4^Department of Statistics, Seoul National University, Seoul, Republic of Korea; ^5^Department of Preventive Medicine, Eulji University, Daejeon, Republic of Korea

## Abstract

A number of statistical methods for detecting gene-gene interactions have been developed in genetic association studies with binary traits. However, many phenotype measures are intrinsically quantitative and categorizing continuous traits may not always be straightforward and meaningful. Association of gene-gene interactions with an observed distribution of such phenotypes needs to be investigated directly without categorization. Information gain based on entropy measure has previously been successful in identifying genetic associations with binary traits. We extend the usefulness of this information gain by proposing a nonparametric evaluation method of conditional entropy of a quantitative phenotype associated with a given genotype. Hence, the information gain can be obtained for any phenotype distribution. Because any functional form, such as Gaussian, is not assumed for the entire distribution of a trait or a given genotype, this method is expected to be robust enough to be applied to any phenotypic association data. Here, we show its use to successfully identify the main effect, as well as the genetic interactions, associated with a quantitative trait.

## 1. Introduction

Recent advances in high-throughput genotyping techniques have produced massive volumes of genetic data. Although it is common to analyze single SNP effects extensively, such approaches cannot adequately explain the intricate genetic contributions to complex diseases such as hypertension, diabetes, and certain psychiatric disorders. Consequently there are still large amounts of genetic components that remain unexplained. Gene-gene interaction analysis may be one method to adequately address this missing heritability problem [1].

For case-control studies, which formulate the measures for a binary trait, a number of statistical methods for detecting gene-gene interactions have been proposed. One of the most popular methods is multifactor dimensionality reduction (MDR) [2] that converts a high-dimensional contingency table to a one-dimensional model without raising the issue of sparse cells. Several variants of MDR have been recently developed [3–8], while another approach was developed [9–11] from information theory [12, 13]. More recently, an entropy-based approach which utilizes the relative gain of information, as well as its standardized measure, has also been proposed [14].

However, for quantitative traits such as the blood pressure, body mass index, and patient survival times, relatively few attempts have been made to analyze the genetic interactions. Because many phenotype measures are intrinsically quantitative, and categorizing a continuous trait may not always be straightforward and meaningful, association of gene-gene interactions with an observed distribution of such phenotypes needs to be investigated directly without categorization. To that end, introducing a new statistic is one way to tackle the problem [15]. Extending the MDR algorithm to continuous traits, as in the ways of the generalized MDR (GMDR) and the model-based MDR (MB-MDR), has been proposed [3, 6]. More recently a quantitative MDR (QMDR) was proposed to replace the balanced accuracy metric with a *t*-test statistic [16]. However, these MDR-based approaches may oversimplify the original data to some degree, through classification of phenotypes. An entropy-based approach may well be an alternative model. Entropy is commonly used in information theory to measure the uncertainty of random variables [12, 13], and information gain or mutual information has been shown useful to represent association strengths [17–19]. Although the usefulness of such information theoretical methods is well known, the statistical methods based on this approach for analyzing gene-gene interactions of the quantitative traits are rarely found, with the exception of one specific case [20]. However, the application may also be limited by assuming a normal distribution.

Here, we extend the usefulness of the information concept to quantitative traits by considering nonparametric estimates based on sample-spacing or *m*-spacing [22–25] for the conditional entropy of a quantitative phenotype, based on a given genotype. The challenge, therefore, is to couple a nonparametric entropy estimator to correct and stable information gains. We thus developed the useful information gain standardized (IGS) approach and applied it to datasets composed of several genotypes and the quantitative trait. This approach could be considered an extension of previous work on categorical traits [14] to the quantitative phenotypes. The proposed method, however, does not attempt in any way to classify quantitative phenotypes like other methods, such as variants of MDR but instead handles them directly, providing an intrinsic advantage of removing the chance of misclassification. While previous entropy-based methods of analyzing quantitative traits assumed the shape of its distribution to be normal [20], our method does not need to specify the distribution to estimate the association. Any regular or irregular distribution would not cause any difficulties. Although this is also an advantage of GMDR or QMDR, we propose a method that takes the advantageous characteristics from both of those methods. We also performed extensive simulation studies to compare the powers of the proposed method to QMDR and GMDR, demonstrating its advantage in detection power.

In the following sections, after a brief review of nonparametric entropy estimation, we describe a new method for modeling genetic interactions. A nonparametric entropy estimator is shown to successfully couple with genetic datasets through our modifying work in the Materials and Methods. Application of this information gain standardized (IGS) approach is evaluated for both simulation and real datasets in the Results and Discussions.

## 2. Materials and Methods

### 2.1. Estimation of the Entropy for a Continuous Variable

If *X* is a random vector with probability density function, *f*(*x*), its differential entropy is defined by

(1) $$\begin{array}{c} H\left(f\right)=-\int f\left(x\right)\ln\left(f\left(x\right)\right)dx. \end{array}$$

A well-known approach for estimating a solution to this equation is to use plug-in estimates. In this approach, *f*(*x*) is first estimated using a standard density estimation method such as a histogram or kernel density estimator, and the entropy is then computed. Integral, resubstitution, splitting data, and cross-validation estimates are among the usual plug-in estimates [22]. Another approach is based on sample-spacing. Let {*X*_*k*_} be a set of independent and identically distributed real valued random variables, with corresponding order statistics of {*X*_*n*,*k*_}. Here, *n* represents the total number of measured samples. For the arbitrary integers *i* and *m* satisfying the condition of 1 ≤ *i* < *i* + *m* ≤ *n*, a spacing of order *m* or *m*-spacing is defined as *X*_*n*,*i*+*m*_ − *X*_*n*,*i*_. A density estimate, based on sample-spacing, *m*, is then constructed as

(2) $$\begin{array}{c} f_{n}\left(x\right)=\frac{m}{n}\frac{1}{X_{n,im}-X_{n,\left(i-1\right)m}}, \end{array}$$

where *x* ∈ [*X*_*n*,(*i* − 1)*m*_, *X*_*n*,*im*_ [14]. This density estimate is consistent if, as *n* → *∞*, *m* → *∞* and *m*/*n* → 0 [22]. Several variations of an entropy estimator with minor differences have been proposed, all based on the above density estimates [23, 24]. Among them, the following were reported to approximate with lowered variance [25]:

(3) $$\begin{array}{c} H_{m,n}=\frac{1}{n-m}\overset{n-m}{\underset{k=1}{\sum }}\ln\left(\frac{n}{m}\left(X_{n,k+m}-X_{n,k}\right)\right). \end{array}$$

Asymptotic bias of this estimator can be corrected by adding additional terms, including the digamma function [22, 28]:

(4) $$\begin{array}{c} H_{m,n}=\frac{1}{n-m}\overset{n-m}{\underset{k=1}{\sum }}\ln\left(\frac{n}{m}\left(X_{n,k+m}-X_{n,k}\right)\right)-\frac{\Gamma^{\prime }\left(m\right)}{\Gamma \left(m\right)}+\ln m. \end{array}$$

As *m* increases, the correctional terms become negligible and the two estimators coincide. Our evaluation of the entropy of a phenotype, *H*(*P*), of a quantitative trait is based on this estimator.

### 2.2. Modification of the *m*-Spacing Based Entropy Estimator

**Figure 1:**
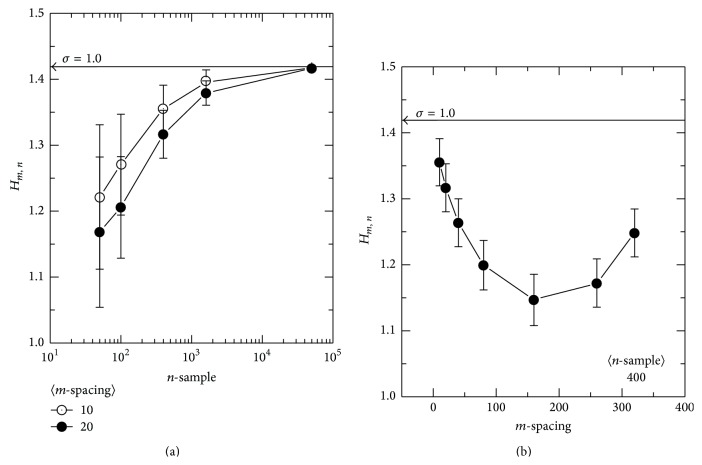
The *n*-dependence (a) and *m*-dependence (b) of the entropy estimator *H*_*m*,*n*_. An ensemble of 3,000 sets of random sampling from *N*(0, 1^2^) was constructed and used for each point in the plot. The sample-spacing, *m*, was fixed while varying the number of samples, *n*, (a) to evaluate the *n*-dependence of the entropy estimator. In (b), *n* was fixed and *m* was varied to show the *m*-dependence. Analytically obtained true values are represented by the arrowed horizontal lines.

The estimator in (4) has both *n* and *m* as parameters. In genetic association studies, the number of samples, *n*, of several hundreds is common. However, when the conditional entropy is estimated, there may be a minor allele that could have a much smaller number of samples corresponding to that allele. Moreover, the choice of the sample-spacing, *m*, should affect the resulting estimation of an entropy value. Therefore, it is required to have an entropy estimation scheme independent of the number of samples, without the need of choosing a particular value of the sample-spacing. To illustrate such a requirement, an ensemble of 3,000 sets of the random deviation from *N*(0, 1^2^) was generated for each data point in Figure 1, where the mean and standard deviation of the estimates are plotted for each ensemble. On the left panel of Figure 1, *m* is fixed to 10 and 20 while *n* is varied. The analytic formula of the entropy for a normal distribution can be obtained as follows [20], where *e* is Euler's number:

(5) $$\begin{array}{c} H=\ln\left(\sigma \sqrt{2\pi e}\right). \end{array}$$

The calculated value of (5) is pointed on the vertical axis with a horizontal arrow with the corresponding *σ* above it. The obvious *n*-dependence of the estimator can be seen in this plot, where the estimation approaches the analytic value, as *n* increases with $\sqrt{n}$-consistency, as expected [24]. In Figure 1(b), *n* is fixed to 400, while *m* is varied. In this plot, the estimated entropy again changes in value throughout the possible range of *m*. It is shown that the estimated value is always smaller than the analytically calculated value. Therefore, assigning a particular value to *m* such as $\sqrt{n}$, the typical choice [25], would not be appropriate in this sampling range. Because of these *n*- and *m*-dependences, the estimator in (4) may need to be modified. Therefore, we modify the entropy estimator in (4) as follows:

(6) Hm,n=1n−1∑m=1n−11n−m∑k=1n−mln⁡nmXn,k+m−Xn,kaaaaaaaaaaaaaaaa−Γ′mΓm+ln⁡m.

**Figure 2:**
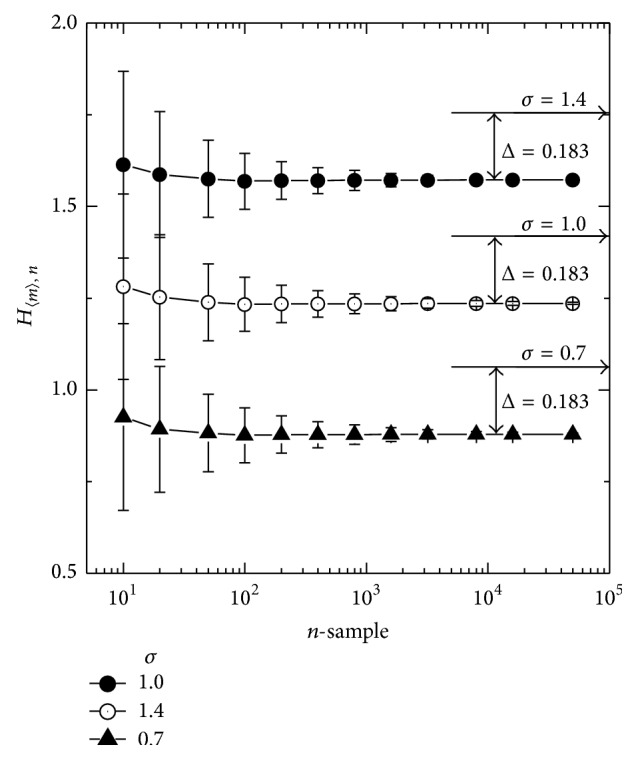
The *n*-independence and constant offset from the true value of the estimates averaged over all possible *m* values for each *n*. Each symbol represents a result of samplings from *N*(0, *σ* ^2^). While varying *n*, the number of samples, the estimated entropy values were averaged over all the possible *m*, sample-spacing values. 〈*m*〉 denotes this averaging, which should not depend on weighting due to the virtually same standard deviations shown in Figure 1(b). Over a wide range of *n*, the estimated entropy stays effectively the same, showing *n*-independence in the range of practical number of sampling. Moreover, the almost flat line connecting each symbol shifts up or down following exactly the change of the true value indicated by the horizontal arrows. The rise in the extremely small *n* range should be within the tolerable error of any specific application, because the contribution to conditional entropy by such a case would be suppressed by weighting, based on the marginal probability that should be proportional to *n*.

In this modification, an entropy estimator is averaged over the possible *m* values for each *n*, which is denoted by 〈*m*〉. This estimator is used to plot the entropy versus number of samples in Figure 2. Over a wide range of *n*, this entropy estimator yields very stable values, in contrast to Figure 1(a). An increase in the extremely small *n* range should be within the tolerable error in an application of genome-wide association, as the contribution to the conditional entropy by such a minor allele would be suppressed by the weighting factor of the marginal probability that should be proportional to the number of corresponding samples. Analytically obtained entropy values for *N*(0, *σ* ^2^), with three different *σ*'s, are marked on the vertical axis on the right-hand side. Regardless of the value of *σ*, the differences between the analytically obtained value and the values given by the estimator stay essentially the same. Considering that the association study measures the difference between the entropy and the corresponding conditional entropy, the stability should be a more critical issue than the absolute value of the estimates. Therefore compensation of this Δ would not be necessary as long as it is stable. Furthermore, the underestimation of the entropy shown in the plot should have little effect on the association strength. Hence, an entropy estimator has been set up that should satisfy the practical *n*-independence without the need to find a proper sample-spacing.

### 2.3. Evaluation of a Conditional Entropy

Now let *G* be a categorical variable assigned to each sample measurement *X*_*k*_. *G* may be a genotype given by a measured SNP or a combination of SNPs, while *X*_*k*_ represents the measured value of a phenotype. For detecting the main effect of a single SNP, *G* consists of three categories of *G* = 0, *G* = 1, and *G* = 2. For detecting the interaction between SNP_*i*_ and SNP_*j*_, *G* consists of 9 categories, such that *G* = 0 = (SNP_*i*_ = 0,  SNP_*j*_ = 0), *G* = 1 = (SNP_*i*_ = 0,  SNP_*j*_ = 1), *G* = 2 = (SNP_*i*_ = 0,  SNP_*j*_ = 2), *G* = 3 = (SNP_*i*_ = 1,  SNP_*j*_ = 0),…, and *G* = 8 = (SNP_*i*_ = 2,  SNP_*j*_ = 2). Detection of the higher order interaction can be performed in the same way with expansion of the categories of *G*. Then an estimator for each specific component of the conditional entropy, *H*(*P*∣*G* = *g*), can be constructed using the genotype-selected subset measurements {*X*_*n*_*g*_,*k*_}, along with an individual sample-spacing of *m*_*g*_. Extending (6), while applying the above argument, should now readily produce the estimators for the entropy of a phenotype and the conditional entropy. Here *d* denotes the order of a gene-gene interaction:

(7) HP=1n−1∑m=1n−11n−m∑k=1n−mln⁡nmXn,k+m−Xn,kaaaaaaaaaaaaaaaaa∑k=1n−mln⁡nmXn,k+m−Xn,k−Γ′mΓm+ln⁡m,HP ∣ G=∑g=03d−1ngn1ng−1∑mg=1ng−11ng−mg∑k=1ng−mgaaaaaaaaaaaaaaaaaaa·∑k=1ng−mgln⁡ngmgXng,k+mg−Xng,kaaaaaaaaaaaaaaaaaaa∑k=1ng−mg−Γ′mgΓmg+ln⁡mg=∑g=03d−1ngnHP ∣ G=g.

### 2.4. Standardized Measure of an Association Strength

Since the differential entropy values are scale-dependent, when the above estimators are calculated with {*X*_*i*_} and {*cX*_*i*_} (where *c* is a constant scale factor), the difference would be ln⁡*c*:

(8) $$\begin{array}{c} H_{\left\{cX_{i}\right\}}=\ln c+H_{\left\{X_{i}\right\}}. \end{array}$$

For example, if the phenotype is height it may be measured in meters or centimeters. In this case, the scale factor is 100. Nevertheless, the association strength should also be the same. Also note that a negative value is perfectly legitimate for a differential entropy. Information gain, IG, as in the form defined with discrete entropies [14], should satisfy scale independence, while correctly representing an association strength without being affected by negative values. Therefore, it should retain its usefulness as a measure of an association strength:

(9) $$\begin{array}{c} \text{IG}=H\left(P\right)-H\left(P\;|\;G\right). \end{array}$$

IG would be readily estimated with the above estimator (7). IG standardized (IGS) is set up with the means and standard deviations of IGs obtained from repeated shuffling of the phenotypes while all genotypes remained fixed [14]. Let IG_*i*_^(1)^ denote the maximum IG of the *i*th permuted dataset. Then, the mean and standard deviation of IG_1_^(1)^, IG_2_^(1)^,…, IG_*n*_^(1)^ can be computed as follows:

(10) $$\begin{array}{c} {\bar{\text{IG}}}_{p}=\frac{\sum_{i=1}^{n}{\text{IG}}_{i}^{\left(1\right)}}{n},\;\;S_{p}=\sqrt{\frac{\sum_{i=1}^{n}{\left({\bar{\text{IG}}}_{i}^{\left(1\right)}-{\bar{\text{IG}}}_{p}\right)}^{2}}{n-1}}, \end{array}$$

where *n* is the number of permuted datasets. Now IGS is defined as follows:

(11) $$\begin{array}{c} \text{IGS}=\frac{\text{IG}-{\bar{\text{IG}}}_{p}}{S_{p}}. \end{array}$$

## 3. Results and Discussions

### 3.1. Demonstration of the *m*-Spacing Method

**Figure 3:**
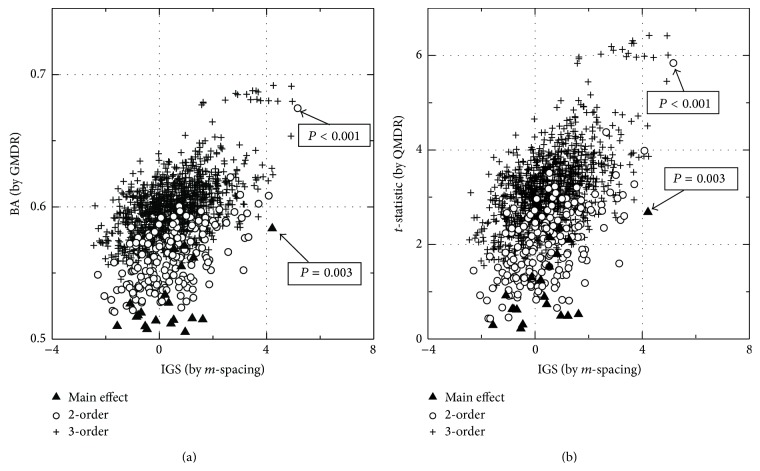
Comparison of the QMDR, GMDR, and *m*-spacing methods. Association strengths obtained by GMDR versus *m*-spacing (a) and by QMDR versus *m*-spacing (b) are compared for a simulated dataset. All three methods were used to evaluate the main effect as well as 2nd and 3rd order interactions. The dataset was designed to have one 2nd order interaction causal pair.

To show the plausibility of the proposed *m*-spacing method, a representative result is shown in Figure 3, using a dataset whose quantitative trait was generated from a normal distribution with a single causal SNP pair simulated, as described in the next section. The sample size of the dataset was 400, with 20 SNPs. In panel (a), the association strengths, obtained by *m*-spacing and GMDR, are plotted as horizontal and vertical coordinates, respectively. Filled triangles represent the main effects, while open circles are for the 2nd order interactions. Both methods identify the same single SNP pair having a prominent interaction plotted in the upper right corner. One of the SNPs was found to produce the main effect, in contrast to others. Again, the result is agreed by both methods. *P* values obtained by permutation are given in the boxes for those selected points. Association strengths of the 3rd order interactions are plotted with a plus sign. Because no 3rd order interaction is simulated into the dataset, the combinations of SNPs made by adding a single SNP to the causal pair are expected to have high association values. Those points are clustered near the identified causal pair in the upper right corner. In panel (b) of Figure 3, the same comparison was made using the result from *m*-spacing and QMDR. Both comparisons show consistent results between the proposed *m*-spacing method and GMDR or QMDR. Note that IGS instead of IG was used. The distribution of the IG values from a dataset would shift to a higher direction, with increased order of interactions. Thus, the more conditions applied, the less entropy may be left to find. In other words, as the order of interaction increases, the conditional entropy *H*(*P*∣*G*) tends to decrease, while *H*(*P*) remains the same. Therefore IGS is vital if one needs to compare the association strengths between genotypes from different orders of interactions. Figure 3 shows that the simulated causal pair has the largest IGS value among all points, from different orders of interactions.

### 3.2. Generation of the Simulation Data

To examine the performance of the *m*-spacing method, an extensive set of simulation data was necessarily generated. First, three types of quantitative trait distributions were considered. Two of them were normal and gamma distributions, and another one was a mixture of those two types. With single causal pair designed, 70 different penetrance models, based on [21], were incorporated. For the case of a normal distribution, a phenotype value, *y*, associated with two interacting SNPs was selected from a normal distribution, as defined by the penetrance values tabled for possible combinations of genotypes associated as follows:

(12) $$\begin{array}{c} y\;|\;\left({\text{SNP}}_{1}=i,{\text{SNP}}_{2}=j\right)\sim N\left(f_{ij},\sigma^{2}\right). \end{array}$$

**Figure 4:**
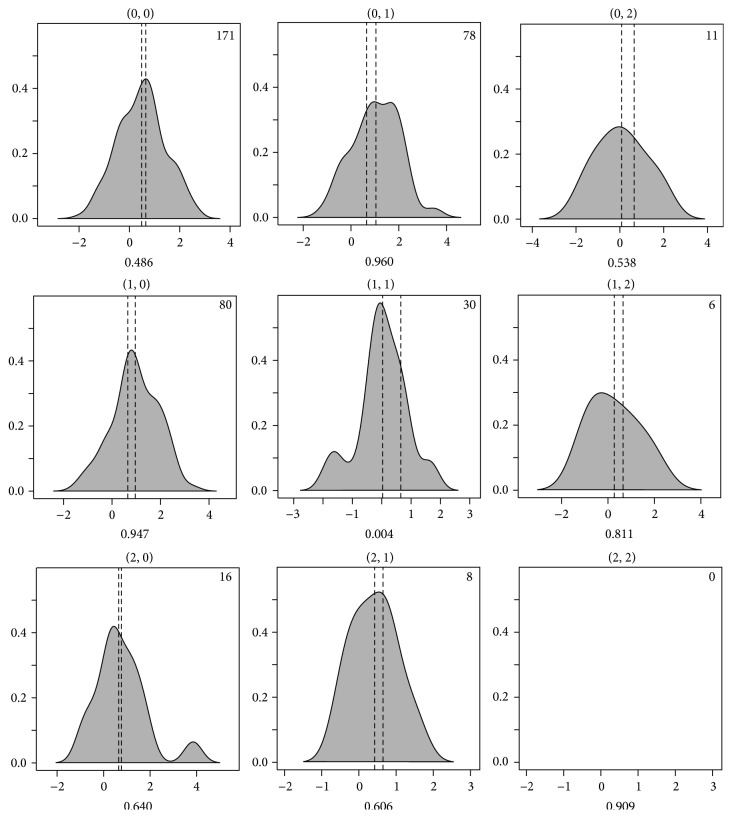
Demonstration of the simulation scheme. Phenotype distributions were plotted to associate with the genotypes by two interacting SNPs, as denoted in the parentheses on top of each plot. SNPs may take values of 0, 1, and 2 or AA, Aa, and aa. For this particular dataset, the MAF was set to 0.200. On the bottom of each plot, the penetrance value for this particular model is given, which is taken from [21]. Inside each plot, the number of samples generated to satisfy the simulation constraint is given. The vertical dotted lines are for the mean values of the high- and low-risk groups. By constraint, the line on the left is for the low-risk group.

Here *f*_*ij*_ represents the penetrance values tabled for every model simulated and can be found in [21]. It is tabulated for each possible pair of genotypes, (*i*, *j*). In 70 different penetrance models, 14 different combinations of two different minor allele frequencies (MAFs) and seven different heritability values were considered. Specifically, we considered the cases when the MAFs were 0.2 and 0.4 and when the heritability was 0.01, 0.025, 0.05, 0.1, 0.2, 0.3, and 0.4. Three different values (0.8, 1.0, and 1.2) of the variance, *σ*, were used independently for the high- and low-risk groups, resulting in 9 combinations. The grouping constraint for the generated event was set such that the averaged *y* of the high-risk group should be larger than or equal to the overall average. The averaged *y* of the low-risk group should be less than the overall average. In Figure 4, 9 possible distributions of a generated phenotype are shown. In this example, the sample size is 400. The high- and low-risk groups have the same number of samples and both have a variance of 1.0. For gamma distributions, phenotype values follow the rule below:

(13) $$\begin{array}{c} y\;|\;\left({\text{SNP}}_{1}=i,{\text{SNP}}_{2}=j\right)\sim \Gamma \left(k,\theta \right). \end{array}$$

The shape and scale parameters, *k* and *θ*, were determined by *f*_*ij*_ and *σ*, using the relationship *f*_*ij*_ = *kθ* and *σ* = *kθ* ^2^. Penetrance models were classified by 7 heritability values: 0.01, 0.02, 0.05, 0.1, 0.2, 0.3, and 0.4, resulting in 10 models for each heritability. The generated data files had a sample size of 400, with 20 SNPs. In all, 3 × 70 × 9 = 1,890 different conditions were set up, with 100 simulated data files generated for each condition.

### 3.3. Comparison of the Detection Probability and Type I Error

**Figure 5:**
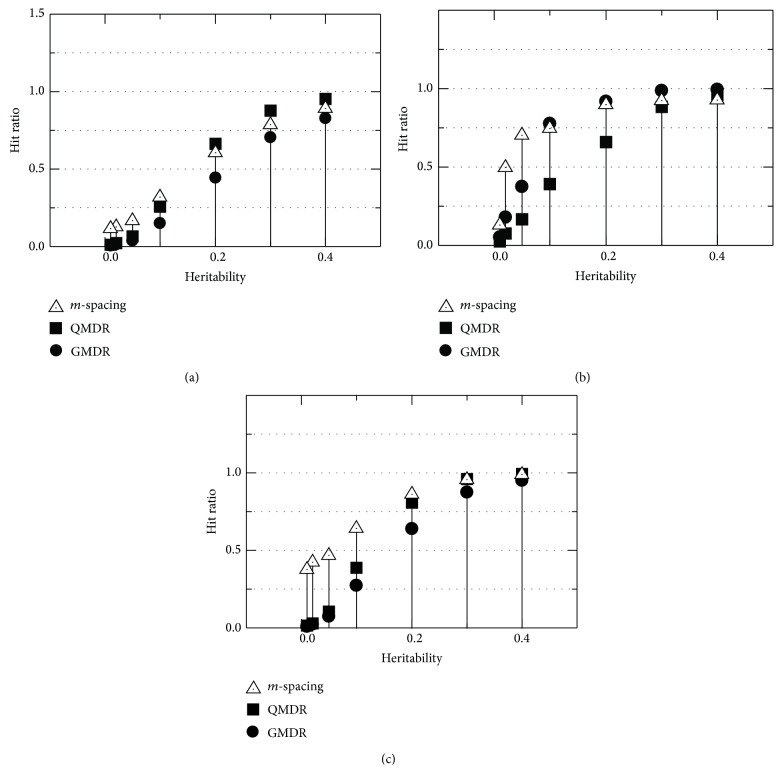
Comparison of the hit ratios or the detection probabilities among the proposed *m*-spacing method, QMDR, and GMDR. Genomic datasets were generated based on 70 different penetrance functions [21], which were, in turn, classified into 7 distinct values of heritability. For each model, the phenotype values are simulated with normal (a), gamma (b), and mixed (c) distributions. High- and low-risk groups in a quantitative trait overlapped with 9 different combinations of the standard deviations. Considering all of the above, 100 data files were generated for each case, adding up to 9,000 simulated files being examined for each point in the plot.

The “hit ratio,” or detection power, of the IGS was evaluated and compared. Simulated data files described in the previous subsection were used. All of them had a single causal pair to identify. In addition to our proposed *m*-spacing method, QMDR and GMDR were used to compare the results.  Figure 5 shows the comparison. Panels (a), (b), and (c) are for the quantitative trait of normal, gamma, and mixed distributions, respectively. Seventy penetrance models were grouped into 7 cases of heritability on the horizontal axis, while all 9 combinations of the variances in high- and low-risk distributions were merged into each heritability case. With a normal distribution, as shown in Figure 5(a), the *m*-spacing's performance was in between those of QMDR and GMDR for higher values of penetrance. However, in the range of penetrance less than 0.2, the *m*-spacing performs best. Note that the QMDR shows higher detection probability than the GMDR throughout the range. In the case of a gamma distribution, as shown in Figure 5(b), the QMDR's performance drops rapidly, as the heritability decreases when the hit ratios of *m*-spacing, as well as the GMDR, stay better than that of QMDR and are comparable to each other. Note the switch of the GMDR and QMDR's performance ranks with the change of the phenotype distribution. What QMDR does is essentially the dichotomization of the observed values of the quantitative phenotypes. Therefore, it should do better with well-defined symmetric distributions, such as a normal distribution, than with an asymmetric one (e.g., gamma distribution). The proposed *m*-spacing method is expected to be effective regardless of the shape of the phenotype distribution, because it makes no assumptions regarding the distribution and is therefore nonparametric, as demonstrated in Figures 5(a) and 5(b). This nonparameterization is again confirmed in Figure 5(c), showing that *m*-spacing outperforms the QMDR and the GMDR, throughout the whole range of heritability, in the case of the mixed form of phenotype distribution. Among the three methods examined, *m*-spacing was the most robust, performing consistently within the range of conditions for the simulation.

To estimate the type I error rate, the null datasets were generated under the same scheme as used for the detection power analysis except that there was no causal pair intended. Now there are 20 SNPs that none of the pairs are expected to have an association. Permutation *P* values for a particular pair were obtained by permuting each dataset 1000 times. We took the significance level *α* as 0.05 to get the ratio of the permutation *P* values smaller than or equal to *α*. We report this ratio as the type I error rate in Table 1, whose accuracy to one decimal place when expressed in percent was ensured by the number of the permutation. Table 1 presents the type I error rate for each combination of three trait distributions, two MAFs, and seven heritability values, along with the overall estimates. Throughout these conditions, the type I error rates are gathered tightly around 5% with maximum and minimum of 5.4% and 4.3%, respectively. Moreover there exists no sign of the dependence on the trait shape, heritability, and MAF. Therefore our proposed method preserved the type I error rates on these conditions.

### 3.4. Application to Real Data

A full-scale real dataset from the Korean Association Resource (KARE) project [20] was analyzed to investigate the effectiveness of the *m*-spacing method. Among the available phenotypes, “height” was chosen with a sample size of 8,842 from the population-based cohort. The total number of SNPs was 327,872, spanning over 22 chromosomes. The “height” phenotype showed to be close to a normal distribution such that the *m*-spacing method may not take advantage of the shape of the phenotype distribution, as discussed in the previous subsection.  Table 2 lists the SNPs, selected by the *m*-spacing method (IGS), that had the strongest main effects. Out of 10 selected SNPs, rs2079795 and rs6440003 coincide with two previous reports [26, 27], although two more matched SNPs, rs11989122 and rs1344672, could be found as results of our analysis using the same tool as in [26], but using the newly imputed dataset. *P* values were estimated by permutation of the phenotype values to make null distributions. Permutations were iterated 100,000 and 10,000 times for the main effect and the interaction, respectively. A clear distinction between rs11989122 and the other selected SNPs can be seen in the IGS values. In Table 3, the 2nd order gene-gene interaction result is given. The top selected pair (rs6499786, rs1788421) was found to have the strongest association with “height,” but the distinction was not so obvious, compared to the case of the main effect.

## 4. Conclusion

In this paper, we present a modified *m*-spacing method for genome-wide association studies with a quantitative trait. The robustness of this method makes it useful for a wide range of sample sizes, while the original *m*-spacing method yields a reliable result only for datasets with a large sample size. Extensive simulation was performed to produce the datasets with different shapes of phenotype distributions, while varying the penetrance functions and adjusting the heritability as well. Causal pair detection probability was unaffected the most by the compared methods, based on the distribution shape and heritability, while GMDR and QMDR showed more dependency. The proposed *m*-spacing method is proven to outperform the others regardless of the shape of the trait distribution and also the range of lower heritability. In the higher heritability region, the performance of the proposed method is comparable to that of GMDR or QMDR, whichever shows better performance in that region. This would lead to versatile applicability of our nonparametric method for quantitative traits, with various characteristics. We applied this method to successfully identify the main effect and gene-gene interactions for the phenotype “height” with the full set of KARE samples. Although several of them overlapped with a previous report, new interactions were also found. Because “height” is presumed to be a trait with a normal distribution having a higher heritability, our method may be said to have performed successfully with no advantage over other methods. More extensive study is needed for quantitative traits, having various characteristics, to further demonstrate the expected robustness of our modified *m*-spacing method.

## Figures and Tables

**Table 1:** Type I error estimation with the significance level *α* of 0.05.

Type I error rate (%)		Normal	Gamma	Mixed
MAF	0.2	5.0	5.0	5.1
	0.4	5.1	5.0	5.1

Heritability	0.01	5.3	5.0	4.8
	0.02	4.9	5.4	5.2
	0.05	5.3	4.3	5.3
	0.1	5.0	5.3	5.1
	0.2	5.0	5.3	5.1
	0.3	4.8	4.9	4.8
	0.4	5.1	4.7	5.3

Overall		5.0	5.0	5.1

**Table 2:** Application of the *m*-spacing method to a full set of KARE samples with the phenotype “height;” main effect.

Main effect				
rs ID	Chromosome	IGS	*P* value	Previous report
rs11989122	8	11.3892	1 × 10^−5^	^*^5.89 × 10^−6^
rs7316119	12	8.7531	1 × 10^−5^	—
rs936634	18	8.6125	2 × 10^−5^	—
rs7632381	3	7.8235	1 × 10^−5^	—
rs2079795	17	7.6542	1 × 10^−5^	2.92 × 10^−6^ Ref. [26]
rs1344672	3	7.6177	1 × 10^−5^	^*^5.21 × 10^−7^
rs2523865	6	7.6044	4 × 10^−5^	—
rs3790199	20	7.5362	2 × 10^−5^	—
rs6440003	3	7.5231	1 × 10^−5^	3.87 × 10^−7^ Ref. [27]
rs17628655	19	7.5117	6 × 10^−5^	—

^*^Identified using the same method as [26] but with imputed data, which is the same one we analyzed.

**Table 3:** Application of the *m*-spacing method to a full set of KARE samples with the phenotype “height;” 2nd order interaction.

2nd order interaction					
rs ID	Chromosome	rs ID	Chromosome	IGS	*P* value
rs6499786	16	rs1788421	21	4.6197	1 × 10^−4^
rs2529232	7	rs1788421	21	4.3869	1 × 10^−4^
rs2241704	19	rs1788421	21	4.3855	1 × 10^−4^
